# Retraction Note: Polyphyllin I suppresses the formation of vasculogenic mimicry via Twist1/VE-cadherin pathway

**DOI:** 10.1038/s41419-025-08274-9

**Published:** 2025-11-27

**Authors:** Ting Xiao, Weilong Zhong, Jianmin Zhao, Baoxin Qian, Huijuan Liu, Shuang Chen, Kailiang Qiao, Yueyang Lei, Shumin Zong, Hongzhi Wang, Yuan Liang, Heng Zhang, Jing Meng, Honggang Zhou, Tao Sun, Yanrong Liu, Cheng Yang

**Affiliations:** 1 High-throughput Molecular Drug Screening Centre, Tianjin International Joint Academy of Biomedicine, 300070 Tianjin, China; 2 State Key Laboratory of Medicinal Chemical Biology and College of Pharmacy, Nankai University, 300000 Tianjin, China; 3 Pathology Department, Shun Yi District Hospital, 101300 Beijing, China; 4 Department of Gastroenterology and Hepatology, Tianjin Key Laboratory of Artificial Cells, Tianjin Institute of Hepatobiliary Disease, Tianjin Third Central Hospital, 300170 Tianjin, China; 5 School of Life Sciences, Nankai University, 300000 Tianjin, China; 6 Drug Safety Evaluation Center, Tianjin International Joint Academy of Biomedicine, 300070 Tianjin, China

Retraction to: *Cell Death and Disease* 10.1038/s41419-018-0902-5, published online 05 September 2018

The Editors-in-Chief have retracted this article. A number of image integrity issues were raised after publication. Specifically:

- Two panels representing different conditions in figure 2A appear to be duplicated with frameshift
- Two PLC panels in supplementary figure 3B appear to be duplicated with frameshift
- The Hep3B Ctrl panel, and the Bel7402 VEGF-a+PPI panel in supplementary figure 3B appear to be duplicated
- A HepG2 VEGF-a panel and a Hep3B VEGF-a panel in supplementary figure 3C appear to be duplicated with frameshift
- The original images provided for figure 6C representing different conditions appears to contain a duplication

The Editors-in-Chief therefore no longer have confidence in the integrity of the data in this article.

Authors Cheng Yang and Tao Sun disagree with this retraction. All remaining authors did not respond to correspondence from the Publisher about this retraction.

